# Respiratory viruses and postoperative hemodynamics in patients with unrestrictive congenital cardiac communications: a prospective cohort study

**DOI:** 10.1186/s40001-023-01003-y

**Published:** 2023-01-20

**Authors:** Kelly C. O. Abud, Clarisse M. Machado, Lucy S. Vilas Boas, Nair Y. Maeda, Eloisa S. Carvalho, Maria Francilene S. Souza, Paula V. Gaiolla, Claudia R. P. Castro, Juliana Pereira, Marlene Rabinovitch, Antonio Augusto Lopes

**Affiliations:** 1 Heart Institute (InCor), University of São Paulo School of Medicine, São Paulo, Brazil; 2 Virology Laboratory, Institute of Tropical Medicine, University of São Paulo School of Medicine, São Paulo, Brazil; 3 Pró-Sangue Foundation, São Paulo, Brazil; 4 Laboratory of Medical Investigation on Pathogenesis and Targeted Therapy in Onco-Immuno-Hematology (LIM-31), University of São Paulo, São Paulo, Brazil; 5 Division of Pediatric Cardiology, Department of Pediatrics, Stanford University School of Medicine, Stanford, CA USA

**Keywords:** Congenital heart disease, Respiratory viruses, Pulmonary hypertension, Pediatric cardiac surgery, Postoperative inflammatory response, Pediatric intensive care

## Abstract

**Background:**

Pulmonary vascular abnormalities pose a risk for severe life-threatening hemodynamic disturbances following surgical repair of congenital cardiac communications (CCC_s_). In the distal lung, small airways and vessels share a common microenvironment, where biological crosstalks take place. Because respiratory cells infected by viruses express a number of molecules with potential impact on airway and vascular remodeling, we decided to test the hypothesis that CCC patients carrying viral genomes in the airways might be at a higher risk for pulmonary (and systemic) hemodynamic disturbances postoperatively.

**Methods:**

Sixty patients were prospectively enrolled (age 11 [7–16] months, median with interquartile range). Preoperative pulmonary/systemic mean arterial pressure ratio (PAP/SAP) was 0.78 (0.63–0.88). The presence or absence of genetic material for respiratory viruses in nasopharyngeal and tracheal aspirates was investigated preoperatively in the absence of respiratory symptoms using real-time polymerase chain reaction (kit for detection of 19 pathogens). Post-cardiopulmonary bypass (CPB) inflammatory reaction was analyzed by measuring serum levels of 36 inflammatory proteins (immunoblotting) 4 h after its termination. Postoperative hemodynamics was assessed using continuous recording of PAP and SAP with calculation of PAP/SAP ratio.

**Results:**

Viral genomes were detected in nasopharynx and the trachea in 64% and 38% of patients, respectively. Rhinovirus was the most prevalent agent. The presence of viral genomes in the trachea was associated with an upward shift of postoperative PAP curve (*p* = 0.011) with a PAP/SAP of 0.44 (0.36–0.50) in patients who were positive versus 0.34 (0.30–0.45) in those who were negative (*p* = 0.008). The presence or absence of viral genomes in nasopharynx did not help predict postoperative hemodynamics. Postoperative PAP/SAP was positively correlated with post-CPB levels of interleukin-1 receptor antagonist (*p* = 0.026), macrophage migration inhibitory factor (*p* = 0.019) and monocyte chemoattractant protein-1 (*p* = 0.031), particularly in patients with virus-positive tracheal aspirates.

**Conclusions:**

Patients with CCC_s_ carrying respiratory viral genomes in lower airways are at a higher risk for postoperative pulmonary hypertension, thus deserving special attention and care. Preoperative exposure to respiratory viruses and post-CPB inflammatory reaction seem to play a combined role in determining the postoperative behavior of the pulmonary circulation.

## Background

In patients with congenital cardiac communications, pulmonary vascular abnormalities pose a risk for immediate postoperative hemodynamic disturbances which are sometimes severe enough to be considered as life-threatening medical emergencies. Besides, persistent pulmonary hypertension late after operation may affect treatment success [1–3]. The severity of postoperative pulmonary vascular reactivity depends on the degree of preoperative pulmonary vascular remodeling which is generally related to patient age, type of cardiac anomaly and presence of extracardiac syndromes, such as Down syndrome (trisomy 21) [4]. Intraoperative factors play a role: metabolic and electrolyte abnormalities, imbalance between endogenous vasoconstrictors and vasodilators, and systemic inflammatory response to cardiopulmonary bypass (CPB) [5]. Postoperatively, hypoxia and acidosis have been considered as major triggers for pulmonary vasoconstriction [6, 7].

Large (unrestrictive) cardiac communications may be associated with pulmonary overcirculation with variable degrees of pulmonary congestion. Patients may present with congestive heart failure and failure to thrive and are predisposed to recurrent upper and lower respiratory tract infections. Viral infections have been extensively investigated in pediatric patients undergoing cardiac surgery. They were shown to impact on major outcomes, such as duration of postoperative mechanical ventilation, length of intensive care unit and hospital stays, and mortality [8–10]. However, the impact of preoperative exposure to respiratory viruses on the postoperative behavior of the pulmonary circulation has not been examined so far.

Respiratory viral infections may affect pulmonary microcirculation in at least two ways. First, they may cause local alveolar hypoxia, a major stimulus for pulmonary vasoconstriction. Second, infected airway epithelial cells cause cascade expression of proinflammatory cytokines, chemokines and growth factors, thus inducing local inflammation, extracellular matrix reorganization and cell proliferation [11–17]. Because small airways and arteries share the same microenvironment in the lungs [18], the biological events that take place as a result of viral infections may in theory, contribute to both airway and vascular remodeling.

In this study, we investigated the possible role of preoperative exposure to respiratory viruses and post-CPB inflammatory reaction in determining the behavior of the pulmonary circulation following elimination of left-to-right cardiac shunts. We examined that in a prospective pediatric cohort taking into consideration classical risk factors for postoperative pulmonary hypertension as well. We also looked for a possible relationship between the presence/absence of viral genomes in the airways prior to surgery and the level of pulmonary arterial pressure after discharge from the hospital.

## Methods

### Study design, setting and patients

This was a prospective cohort study comprising patients who were admitted to the Heart Institute (InCor), University of São Paulo School of Medicine, São Paulo Brazil, from November 2016 to September 2021, for surgical repair of congenital cardiac communications. Patients were consecutively enrolled based on the following criteria: age 1 month to 3 years; heart with biventricular physiology; presence of unrestrictive cardiac communications, i.e., with a diameter of the post-tricuspid communication greater than 50% of the aortic annulus diameter on transthoracic echocardiography; absence of pulmonary stenosis; clinical features suggestive of at least moderately elevated pulmonary arterial pressure: a loud second heart sound at pulmonic region, increased pulmonary/systemic blood flow ratio with absence of significant pressure gradients across the septal defects (echocardiography) and increased pulmonary vascular markings on chest radiographs; absence of extracardiac syndromes other than Down syndrome; and absence of any signs of ongoing or recent inflammatory or infectious diseases. The study was based essentially on patients who were eligible for surgical treatment with no need for cardiac catheterization. Patients with significant right-to-left shunting and persistently low peripheral oxygen saturation (< 85%), suggesting the presence of advanced pulmonary vasculopathy, were not included. An informed consent signed by family members was necessary for patient inclusion. The study protocol was approved by the Institutional Scientific and Ethics Committee, CAPPesq no. 3.735.675.

### General diagnostic data

Data recorded at baseline included details of the clinical history and physical examination, such as signs and symptoms of congestive heart failure and failure to thrive, presence or absence of Down syndrome, occurrence of acute respiratory infections and eventual hospitalization prior to referral to the Heart Institute. The diagnosis of Down syndrome was confirmed by genetic testing. Chest radiographs, ECG and complete echocardiographic data were available for all patients. Transthoracic echocardiography was used to assess cardiovascular anatomy and blood flow parameters. The pulmonary/systemic blood flow ratio was calculated based on systolic flow parameters in the right and left ventricular outflow tracts. Pulmonary venous flow was estimated by measuring the velocity–time integral of blood flow in pulmonary veins. Patients with a pulmonary blood flow ratio greater than 2.50 and a velocity–time integral of pulmonary venous flow > 24.0 cm were considered to have pulmonary over circulation which was generally associated with normal (> 93%) peripheral oxygen saturation. Subjects with a pulmonary/systemic blood flow ratio < 2.00, a velocity–time integral of blood flow in pulmonary veins < 20.0 cm and peripheral oxygen saturation < 93% were presumed to have heightened pulmonary vascular resistance, thus requiring special attention postoperatively. The tricuspid annular plane systolic excursion (TAPSE) was used to estimate right ventricular systolic function.

### Perioperative management

Patients underwent open cardiac surgery for repair of cardiac lesions. Intraoperatively, they had pulmonary arterial pressure and systemic arterial pressure levels recorded before and after cardiopulmonary bypass (CPB). In our institution, patients who are at risk for postoperative pulmonary hemodynamic disturbances, i.e., those with unrestrictive cardiac communications and preoperative signs of pulmonary hypertension are routinely weaned from CPB on 20 ppm inhaled nitric oxide. A pulmonary arterial catheter was inserted by the surgeon to facilitate hemodynamic monitoring in the intensive care unit. Postoperative analgosedation was performed using fentanyl, midazolan and ketamine singly or in combination. Alternatively, morphine and dexmetomidine were used in combination in subjects with a very stable clinical course. Milrinone, epinephrine and norepinephrine were used as inotropic/vasoactive agents. Postoperatively, patients were kept on inhaled nitric oxide during the entire period of mechanical ventilation, except those in whom ventilation was prolonged for reasons other than pulmonary hypertension.

### Primary outcome: postoperative hemodynamics

Invasive assessment of pulmonary and systemic arterial pressures was carried out for at least 2.5 days postoperatively (readings taken at 2-h intervals). Patients with unstable clinical course required longer periods of hemodynamic monitoring. To analyse differences between the study groups (i.e., patients with versus without viral genomes in the respiratory tract) pressure curves were constructed based on data obtained during the first 12 h of intensive care unit stay. In that period, patients were still deeply sedated, stable on mechanical ventilation receiving 20 ppm inhaled nitric oxide, and already free from major post-CPB hemodynamic instabilities. We also computed pulmonary/systemic mean arterial pressure ratio (PAP/SAP) and calculated the mean of first 4 values corresponding to the first 6 h of postoperative monitoring. In the study, this parameter was referred to as *early postoperative PAP/SAP*. It was also used to compare groups.

### Other assessments during and after hospitalization

Postoperative clinical events related to pulmonary vascular tone instability and acute right/left ventricular dysfunction were recorded. These included: (1) typical pulmonary hypertensive crisis defined as a sustained elevation of pulmonary arterial pressure (mean pulmonary arterial pressure > 75% of systemic arterial pressure level) with a decline in systemic pressure (≥ 20%) and oxygen desaturation (< 90%); (2) systemic hypotension with a pulmonary/systemic mean arterial pressure ratio in the range of 50–75% requiring frequent changes in the doses of vasoactive-inotropic drugs; (3) prolonged and/or recurrent hemodynamic and respiratory disturbances not promptly responsive to sedation and manual ventilation; and (4) all hemodynamic and respiratory critical instabilities requiring cardiorespiratory resuscitation. Transient elevations of pulmonary arterial pressure, even to suprasystemic levels, that were rapidly reversed by sedation and manual ventilation were not characterized as clinical events. Interpretations were made independently by 3 physicians (AAL, AMT, and on-duty intensivist).

After discharge from the hospital patients were followed up for 6 months, and then pulmonary hemodynamics was re-evaluated noninvasively by means of transthoracic echocardiography. An adequate tricuspid regurgitation jet Doppler signal was required to estimate systolic pulmonary arterial pressure. That was not possible in subjects with negligible tricuspid regurgitation. All factors and covariates that were recorded perioperatively were tested for a possible role in predicting post-hospitalization pulmonary arterial pressure.

### Respiratory viruses

Nasopharyngeal and tracheal aspirates were obtained from all patients for detection and identification of respiratory viruses. Nasopharyngeal aspirates were obtained 2–3 days before surgery in the complete absence of respiratory symptoms. Tracheal aspirates were collected in the operating room just after orotracheal intubation. Nucleic acid extraction was performed using the EASYMAG system (bioMérieux, France) according to the manufacturer’s instructions. Samples were subjected to real-time polymerase chain reaction (RT-PCR, one step) using the ABI 7500 system (Applied Biosystems, CA, USA). A Multiplex Kit (XGen, Pinhais, PR, Brazil) was used for detection of 19 respiratory pathogens including 18 viruses and *Mycoplasma pneumoniae*.

### Pre- and postoperative inflammatory profile

Peripheral venous blood was collected 2–3 days before surgery and 4 h after CPB termination for analysis of serum levels of 36 inflammatory mediators. Proteins were analyzed by immunoblotting using a human cytokine array (R&D Systems, Minneapolis, MN, USA). Samples were processed in duplicate and proteins were semiquantified by chemiluminescence. The results were obtained taking the average signal of each pair of duplicate spots and expressed as units of pixel intensity (upi). Preoperative and postoperative samples were always run in the same assay.

### Data obtainment

Preoperative and postoperative clinical and hemodynamic assessments and laboratory analyses (respiratory viruses and inflammatory mediators) were carried out in a blinded fashion.

### Statistical analysis

Unless otherwise specified, numerical data are presented as medians with interquartile ranges. Categorical data are presented as number of cases and percentages. Descriptive statistics was performing using the Mann–Whitney test and the Chi-square family of tests for comparisons between groups. The Wilcoxon test, Friedman’s test and linear regression analysis were used to test for differences and associations within subjects. Inferential statistics was carried out using the General Linear model (one-way GLM analysis and two-way GLM analysis for repeated measures) to test for differences between groups regarding the behavior of hemodynamic parameters and curves. In this case, the distribution of the dependent variables was tested for closeness to the normal (Gaussian) distribution and when necessary, variables were analyzed after Box–Cox transformation. Predictors of hemodynamic abnormalities and clinical events were identified using univariate, bivariate and multivariate Logistic regression analysis. Once a predictor was identified, a second variable was characterized as a confounder or enhancer if its inclusion in the statistical model resulted in a > 10% change in the original odds ratio. In all tests, 0.05 was set as the significant level. Statistical analysis was performed using the SPSS statistical software, version 28 (IBM, Armonk, NY, USA).

## Results

### Patient’s baseline information

**Fig. 1:**
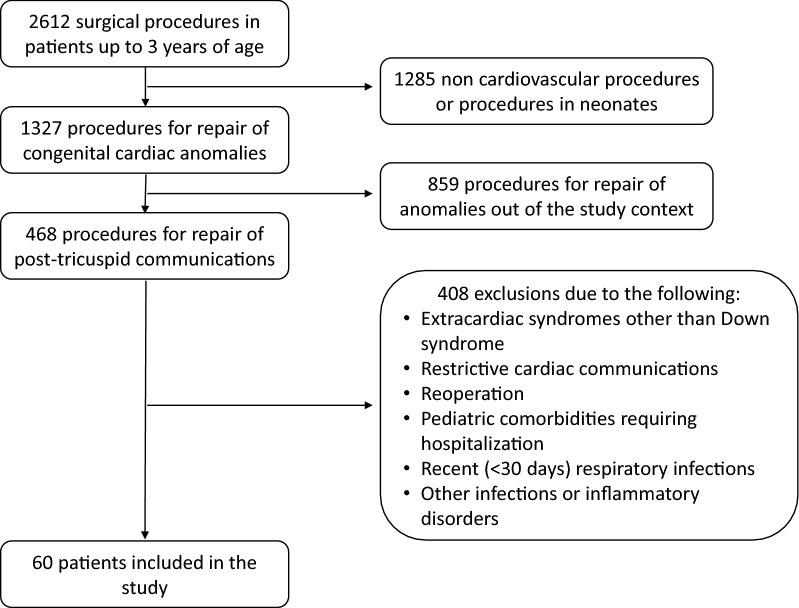
Flow diagram showing patient inclusion and exclusion during the period of the study

A flow diagram showing how patients were selected is shown in Fig. 1. Sixty patients were enrolled with age range of 3–35 months (11 [7–16] months, median with interquartile range). There were 39 patients with Down syndrome, 24 of whom had complete atrioventricular septal defect. All other patients had unrestrictive ventricular septal defect, either isolated (23 cases) or in combination with atrial septal defect, patent ductus arteriosus, mitral, tricuspid or aortic regurgitation, or aortopulmonary window (13 cases). For the entire cohort, pulmonary/systemic blood flow ratio was 2.20 (1.73–2.90) and peripheral oxygen saturation was 96% (93–98%). The presence of relatively restricted pulmonary blood flow considering the size of the cardiac communications suggested that pulmonary vascular resistance was elevated in some cases. Peripheral oxygen saturation was negatively correlated with pre-CPB PAP/SAP (*r* = − 0.34, *p* = 0.009) further suggesting some degree of right-to-left shunting at baseline. On the other hand, 25% of patients had a pulmonary/systemic blood flow ratio greater than 2.90 which was generally associated with overt pulmonary congestion and failure to thrive. According to information obtained from parents, at least 20% of patients had respiratory viral infections prior to referral to the Heart Institute. Twenty-five patients (41.7%) had episodes of bronchiolitis and 22 (36.7%) had pneumonia requiring hospitalization.

### Surgery and immediate postoperative course

All patients underwent complete surgical repair of cardiac anomalies. Pre-CPB mean pulmonary arterial pressure and pulmonary/systemic mean arterial pressure ratio were elevated. Both parameters decreased significantly after elimination of cardiac shunts. However, in the majority of patients they remained above optimal guidelines levels of 20 mmHg and 0.30, respectively [19]. Pre- and post-CPB mean pulmonary arterial pressure was 32 (28–38) mmHg and 21 (19–26) mmHg, respectively, *p* < 0.001. For pulmonary/systemic mean arterial pressure ratio values were 0.78 (0.63–0.88) and 0.40 (0.33–0.48), respectively, *p* < 0.001. Postoperatively, 14 patients had relevant clinical events as defined, which contributed to prolonged mechanical ventilation (*p* < 0.001). One patient died of septicemia. Another patient died in the course of rapid-onset systemic hypotension followed by bradycardia unresponsive to vasopressin and other life-supporting interventions.

### Detection of respiratory viruses

**Fig. 2:**
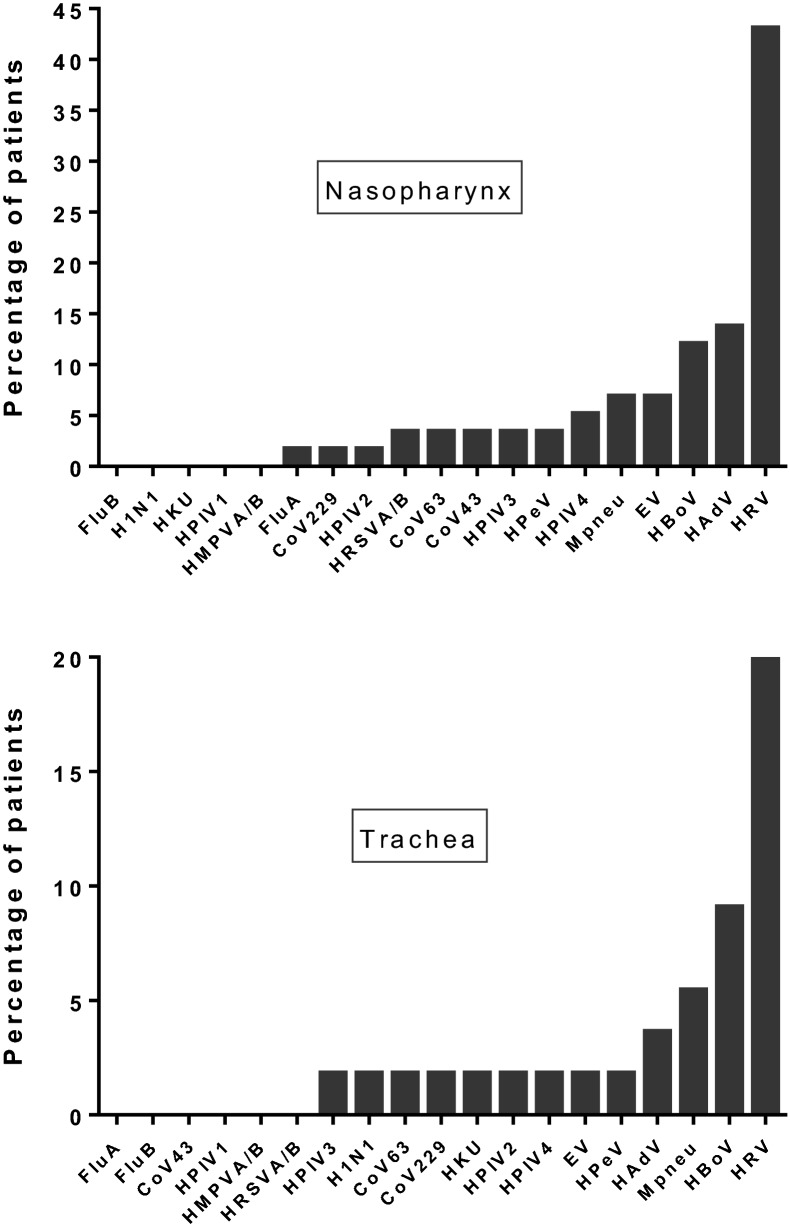
Respiratory viral genomes detected in nasopharyngeal and tracheal aspirates (total of 58 and 55 cases analyzed, respectively). FluA and FluB: influenza A and influenza B, respectively; H1N1: influenza A H1N1; CoV43, CoV63, CoV229 and HKU: coronavirus OC43, NL63, 229E and HKU1, respectively; HPIV1, HPIV2, HPIV3 and HPIV4: human parainfluenza virus 1, 2, 3 and 4, respectively; HMPVA/B: human metapneumovirus A/B; HRSVA/B: human respiratory syncytial virus A/B; HPeV: human parechovirus; EV: enterovirus; HAdV: human adenovirus; HBoV: human bocavirus; HRV: human rhinovirus; Mpneu: *Mycoplasma pneumoniae*

It was possible to characterize respiratory viral genomes in nasopharyngeal and tracheal aspirates in 58 and 55 patients, respectively. For the remaining patients, the amount of molecular material present in the aspirates was considered insufficient for analysis. The percentage of patients carrying genetic material for each of the viruses investigated in the study is shown in Fig. 2. Human rhinovirus was found to be the most prevalent agent in nasopharynx and trachea. Thirty-seven patients (64%) were positive for respiratory viral genomes in nasopharynx, of whom 20, 10, 5 and 2 were carriers for 1, 2, 3 and 4 viruses, respectively. Twenty-one patients (38%) were positive for viral genomes in the trachea, of whom 14, 5 and 2 were carriers for 1, 2 and 3 viruses, respectively. In 1 out of 9 patients who had tracheal aspirates collected and analyzed during the pandemia, SARS-CoV-2-specific RT-PCR test was positive. This patient also tested positive for human parainfluenza virus 3, human bocavirus and *Mycoplasma pneumoniae*.

### Factors influencing postoperative hemodynamics

**Fig. 3:**
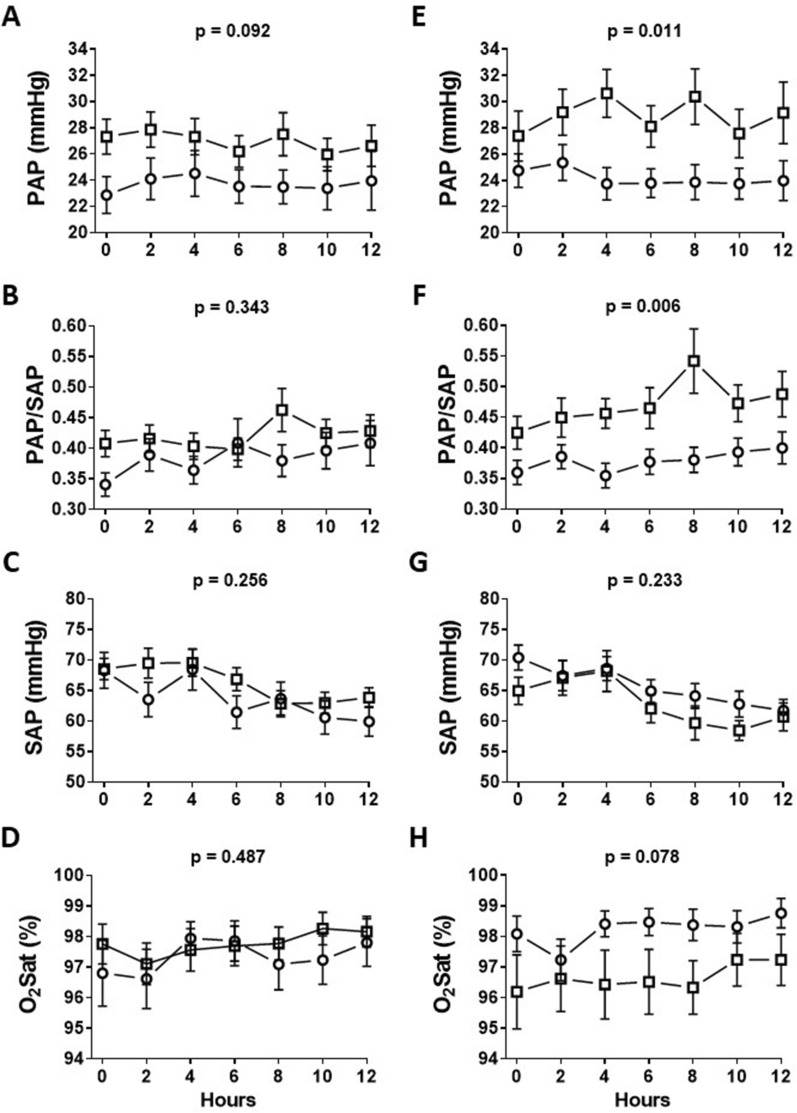
Pressure and oxygen saturation curves during the first 12 h of postoperative care according to the presence (squares) or absence (circles) of respiratory viral genomes in nasopharyngeal (**A**–**D**) and tracheal (**E**–**H**) aspirates. Results are presented as means with SE. *P* values correspond to differences between groups analyzed using the general linear model for repeated measures after Box–Cox transformation of the dependent variables. O_2_ Sat: peripheral oxygen saturation; PAP and SAP: pulmonary arterial pressure and mean systemic arterial pressure, respectively; PAP/SAP: pulmonary/systemic arterial pressure ratio

The behavior of pulmonary and systemic arterial pressure and peripheral oxygen saturation during the first 12 h of postoperative care is shown in Fig. 3. When patients were analyzed according to the presence or absence of viral genomes in the nasopharynx, similar curves were obtained for all parameters. However, patients who were positive for viral genomes in tracheal aspirates had significantly higher pulmonary arterial pressure levels compared to those who were negative. Furthermore, there was a trend toward lower oxygen saturation levels in the former group. Patients with versus without viral genomes in the trachea were subsequently compared in terms of demographic and diagnostic features as well as pre-CPB hemodynamics. No significant differences were observed (Table 1). There were no differences in baseline levels of inflammatory proteins between the groups, except for chemokine SDF-1 whose levels were lower in the former one (respectively, 3569 [2528–4972] and 4998 [3955–6496] units of pixel intensity, *p* = 0.037).

**Table 1:** Demographic and diagnostic data in patient groups

	Viral genomes in tracheal aspirates		
	Absent (*n* = 34)	Present (*n* = 21)	*p* value
Age, months	10 (7–17)	11 (8–15)	0.621^†^
Gender, M:F	14:20	7:14	0.767^‡^
Weight, kg	6.6 (5.4–8.1)	6.4 (6.0–7.5)	0.993^†^
Height, cm	65.0 (61.8–72.5)	66.0 (62.0–70.5)	0.835^†^
Down syndrome, *n* (%)	26 (76.5)	10 (47.6)	0.058^‡^
Peripheral oxygen saturation, %	96 (93–98)	96 (92–98)	0.875^†^
Transthoracic echocardiography			
Main cardiac anomaly, ventricular septal defect: atrioventricular septal defect	19:15	14:7	0.610^‡^
Pulmonary/systemic blood flow ratio	2.25 (1.70–3.05)	2.20 (1.55–2.90)	0.597^†^
Velocity–time integral of blood flow in pulmonary veins, cm^a^	22.4 (18.8–25.3)	21.3 (20.0–25.0)	0.815^†^
TAPSE, mm^§^	15.0 (13.0–17.0)	13.0 (12.0–16.0)	0.155^†^
CPB time, min	132 (90–151)	125 (95–152)	0.979^†^
Pre-CPB mean pulmonary artery pressure, mmHg	32 (28–36)	37 (30–40)	0.070^†^
Pre-CPB pulmonary/systemic mean arterial pressure ratio	0.79 (0.62–0.86)	0.83 (0.68–0.94)	0.212^†^

Numeric variables are presented as median with interquartile range

*CPB* cardiopulmonary bypass

^†^Mann–Whitney test

^‡^Chi-square test

^§^Tricuspid annular plane systolic excursion. Normally, values increase with increasing age in the pediatric population. A TAPSE of ≥ 15.5 is considered normal at the age of 1 year, as are values ≥ 16.5 by the age of 2 years [24]

^a^Values < 20 cm are generally associated with elevated pulmonary vascular resistance in pediatric patients with unrestrictive cardiac shunts [22, 23]

The analysis of factors with possible influence on postoperative hemodynamics in addition to the presence of viral genomes in the trachea was carried out using early postoperative PAP/SAP as dependent variable; values > 0.40 were shown to be associated with relevant postoperative clinical events [20]. In the present study, early postoperative PAP/SAP in patients with and without respiratory viral genomes in tracheal aspirates was 0.44 (0.36–0.50) and 0.34 (0.30–0.45), respectively, *p* = 0.008. Using univariate analysis, we identified 8 factors potentially correlatable with early postoperative PAP/SAP, with *p* values < 0.10 (Table 2). However, only baseline peripheral oxygen saturation, identified as a protective factor (odds ratio 0.40 for quartiles, 95% CI 0.23–0.72, *p* = 0.002) and the presence of viral genomes in the trachea (risk-factor, odds ratio 4.42, 95% CI 1.17–16.78, *p* = 0.029) remained in the multivariate model. The final logistic regression equation was: Ln (*p*/1 − *p*) = 1.290 + 1.487* 0 or 1 for absence or presence of viruses − 0.909* baseline oxygen saturation, where *p* is the probability of having an early postoperative PAP/SAP > 0.40. Because rhinovirus was the most prevalent agent, we analyzed early postoperative PAP/SAP comparatively in patients carrying only rhinovirus (*n* = 8) and those who were negative for all investigated viruses (*n* = 34). The respective values were 0.45 (0.33–0.59) and 0.34 (0.30–0.45) (*p* = 0.041 after adjustment for baseline oxygen saturation). We also analyzed the postoperative behavior of the pulmonary circulation specifically in patients who entered the study prior to SARS-CoV-2 pandemia. Early postoperative PAP/SAP in patients with (*n* = 17) and without (*n* = 29) viral genomes in tracheal aspirates was 0.42 (0.36–0.53) and 0.34 (0.29–0.43), respectively, *p* = 0.007. For patients who entered the study during the pandemia, values were 0.44 (0.36–0.46) (*n* = 4) and 0.35 (0.30–0.57) (*n* = 5), respectively.

**Table 2:** Variables possibly associated with postoperative elevation of pulmonary arterial pressure

	Coefficient	Odds ratio^a^	95% CI		*p* value
			Lower limit	Upper limit	
Age	0.244	1.28	0.81	2.02	0.296
Gender	− 0.454	0.64	0.22	1.86	0.408
Weight	− 0.024	0.98	0.62	1.54	0.920
Height	0.159	1.17	0.75	1.85	0.491
Down syndrome	0.968	2.63	0.85	8.20	0.095
Main cardiac anomaly, AVSD compared to VSD	1.33	3.79	1.28	11.25	0.017
Peripheral oxygen saturation	− 0.701	0.50	0.30	0.81	0.005
Pulmonary/systemic blood flow ratio	− 0.112	0.89	0.56	1.42	0.637
Pulmonary venous flow (VTI)	0.355	1.43	0.90	2.27	0.135
TAPSE	− 0.454	0.64	0.39	1.05	0.076
CPB time	0.931	2.54	1.45	4.44	0.001
Pre-CPB mean pulmonary artery pressure	0.504	1.66	1.01	2.73	0.048
Pre-CPB pulmonary/systemic mean arterial pressure ratio	0.498	1.65	1.01	2.69	0.047
Presence of respiratory viral genomes in the trachea	1.223	3.40	1.09	10.59	0.035

Results were obtained using logistic regression analysis. All numeric variables were analyzed as quartiles

*AVSD* atrioventricular septal defect, *CPB* cardiopulmonary bypass, *TAPSE* tricuspid annular plane systolic excursion, *VSD* ventricular septal defect, *VTI* velocity–time integral of blood flow in pulmonary veins

^a^Associated with early postoperative pulmonary/systemic mean arterial pressure ratio > 0.40 (mean of first 4 values, readings at 2-h intervals)

### Postoperative inflammatory reaction

Changes in hematological parameters from baseline are shown in Table 3. Postoperatively, there was a decrease in lymphocyte and platelet count while monocytes, neutrophils and the neutrophil to lymphocyte ratio increased significantly. The increase in platelet volume was compatible with increased platelet turnover. C-reactive protein was importantly increased on postoperative day 1. Post-CPB systemic inflammatory response was characterized by changes in serum levels of several inflammatory proteins, as shown in Table 4. For example, the decrease in serum RANTES was directly related to the amount of vasoactive and inotropic drugs required for hemodynamic stabilization which was computed using the vasoactive-inotropic score [21] (*r* = 0.50, *p* < 0.001). Besides, there was a marked increase in circulating IL-1RA with negligible changes in IL-1 (spare receptor phenomenon). The relationship between post-CPB inflammatory response and development of postoperative pulmonary hypertension was investigated. Of 36 inflammatory proteins that were analyzed in serum 4 h post CPB, 10 were found to have a possible influence on early postoperative PAP/SAP with *p* values < 0.10 (Table 5). To investigate if respiratory viruses and post-CPB inflammatory reaction could have combined effects on postoperative hemodynamics we adjusted bivariate models for each of the proteins listed in the table using the presence/absence of viral genomes in tracheal aspirates as the second variable. For 3 cytokines, the inclusion of the second variable resulted in a > 10% increase in the odds ratio obtained at univariate analysis, thus suggesting a role for viruses and cytokines as predisposing and triggering factors, respectively (Table 5).

**Table 3:** Preoperative and postoperative hematological data

	Before surgery	Post-CPB		*p* value*
		4 h	24 h	
White blood cell count, K/μL	10.54 (7.69–12.21)	14.23 (11.45–17.02)^†^	13.93 (11.59–18.33)^†^	< 0.001
Lymphocytes, K/μL	4.78 (3.58–7.76)	1.69 (1.00–2.44)^†^	1.77 (1.17–2.19)^†^	< 0.001
Monocytes, K/μL	0.68 (0.51–1.06)	1.05 (0.72–1.43)^‡^	1.44 (0.79–1.88)^†^	< 0.001
Neutrophils, K/μL	3.28 (2.06–4.90)	10.82 (8.68–13.69)^†^	10.70 (8.08–13.66)^†^	< 0.001
Neutrophil to lymphocyte ratio	0.57 (0.38–0.97)	6.88 (4.03–10.66)^†^	6.33 (4.20–9.42)^†^	< 0.001
Platelets, K/μL	328 (265–380)	140 (114–170)^†^	138 (110–163)^†^	< 0.001
Mean platelet volume, fL	9.40 (8.80–10.00)	9.30 (8.60–9.93)	10.00 (9.20–10.50)^†^	< 0.001
C-reactive protein, mg/L	1.88 (1.06–3.30)	2.77 (1.80–4.30)	69.01 (54.40–79.79)^†^	< 0.001

Results are presented as median with interquartile range

*CPB* cardiopulmonary bypass

*Friedman test

† and ‡, respectively, *p* < 0.001 and *p* < 0.01 versus baseline

**Table 4:** Changes from baseline in serum levels of inflammatory proteins following CPB

	Before surgery	4 h post-CPB	Valor de p*
C5/C5a (upi)	3193 (1199–6766)	1796 (1073–3578)	0.021
CD40L (upi)	6148 (3130–10,662)	2474 (1263–4975)	< 0.001
G-CSF (upi)	448 (265–814)	1222 (685–1990)	< 0.001
GM–CSF (upi)	327 (161–440)	279 (171–397)	0.753
GROα (upi)	1947 (1319–3250)	1301 (757–2135)	0.001
I-309 (upi)	505 (254–1158)	623 (300–935)	0.995
ICAM-1 (upi)	41,303 (31,628–52,121)	39,366 (28,739–48,650)	0.398
IFN-γ (upi)	294 (180–407)	285 (187–432)	0.302
IL-1α (upi)	365 (202–617)	366 (227–727)	0.624
IL-1β (upi)	170 (124–470)	224 (122–436)	0.807
IL-1RA (upi)	1626 (730–2450)	15,023 (11,424–20,347)	< 0.001
IL-2 (upi)	192 (120–490)	222 (147–379)	0.666
IL-4 (upi)	337 (166–488)	295 (206–461)	0.369
IL-5 (upi)	126 (79–240)	136 (75–214)	0.630
IL-6 (upi)	301 (180–621)	1198 (563–2128)	< 0.001
IL-8 (upi)	267 (148–447)	482 (231–828)	< 0.001
IL-10 (upi)	250 (141–391)	284 (205–461)	0.050
IL-12p70 (upi)	261 (151–421)	239 (132–413)	0.554
IL-13 (upi)	619 (318–1208)	671 (356–1157)	0.130
IL-16 (upi)	592 (290–1085)	1859 (1171–2840)	< 0.001
IL-17 (upi)	293 (177–477)	273 (175–521)	0.225
IL-17E (upi)	400 (208–639)	350 (239–602)	0.781
IL-27 (upi)	283 (157–415)	251 (158–406)	0.817
IL-32α (upi)	583 (281–977)	442 (284–940)	0.133
IP-10 (upi)	787 (425–1375)	1519 (787–3826)	< 0.001
I-TAC (upi)	519 (279–913)	504 (319–807)	0.959
MCP-1 (upi)	439 (232–824)	676 (378–1028)	0.001
MIF (upi)	5529 (4652–7003)	7609 (6733–9935)	< 0.001
MIP-1α/β (upi)	524 (269–878)	491 (281–921)	0.635
Serpin E1 (upi)	44,650 (34,461–56,664)	36,631 (31,107–47,622)	0.006
RANTES (upi)	61,983 (47,488–71,255)	50,689 (39,347–65,383)	0.004
SDF-1 (upi)	4348 (3333–6127)	4321 (2911–6630)	0.933
TNF-α (upi)	306 (149–585)	304 (203–584)	0.243
sTREM-1 (upi)	280 (177–533)	285 (196–583)	0.462
IL-18 (upi)	1058 (544–1576)	1055 (558–1799)	0.103
IL-21 (upi)	411 (266–732)	414 (282–684)	0.440

Results are presented as median with interquartile range

*CPB* cardiopulmonary bypass

*Wilcoxon test

**Table 5:** Inflammatory markers and postoperative elevation of pulmonary arterial pressure: univariate and bivariate analyses

	Coefficient	Odds ratio^a^	95% CI		*p* value
			Lower limit	Upper limit	
IL-1RA	0.583	1.79	1.02	3.14	0.041
IL-6	0.708	2.03	1.10	3.76	0.024
IL-10	0.599	1.82	1.00	3.31	0.049
IL-17E	0.558	1.75	0.98	3.13	0.061
MCP-1	0.468	1.60	0.92	2.78	0.096
MIF	0.583	1.79	1.02	3.14	0.041
MIP-1α/β	0.558	1.75	0.98	3.13	0.061
TNF-α	0.522	1.69	0.94	3.02	0.079
IL-18	0.599	1.82	1.00	3.31	0.049
IL-21	0.818	2.27	1.23	4.19	0.009
IL-1RA	0.711	2.04 (14% increase)	1.09	3.81	0.026
Presence of respiratory viral genomes in the trachea	1.475	4.37	1.08	17.63	0.038
MIF	0.849	2.34 (31% increase)	1.15	4.74	0.019
Presence of respiratory viral genomes in the trachea	1.432	4.19	1.03	16.96	0.045
MCP-1	0.780	2.18 (36% increase)	1.07	4.43	0.031
Presence of respiratory viral genomes in the trachea	1.843	6.32	1.30	30.71	0.022

Cytokine concentrations were measured 4 h after cardiopulmonary bypass termination and analyzed as quartiles

Shown in bivariate analysis are the proteins with a > 10% increase in odds ratio relative to univariate analysis

The first step consisted of selecting cytokines with a *p* value < 0.10

^a^Associated with early postoperative pulmonary/systemic mean arterial pressure ratio > 0.40 (mean of first 4 values, readings at 2-h intervals). Results were obtained using logistic regression analysis

### Early postoperative hemodynamics and clinical events

Postoperative clinical events were closely related to early postoperative hemodynamics, as shown in Table 6. There was a role for hemodynamic parameters recorded in the operating room as well. Multivariate analysis showed that early postoperative PAP/SAP was positively correlated with the occurrence of postoperative events (odds ratio 8.40 for quartiles, 95% CI 1.79–39.47, *p* = 0.007), while higher levels of post-CPB mean systemic arterial pressure were protective (odds ratio 0.42 for quartiles, 95% CI 0.20–0.89, *p* = 0.023). Importantly, there was a direct relationship between the occurrence of postoperative events and the duration of mechanical ventilation (*p* < 0.001).

**Table 6:** Variables with possible association with postoperative clinical events

	Coefficient	Odds ratio	95% CI		*p* value
			Lower limit	Upper limit	
Age	0.129	1.14	0.67	1.94	0.635
Gender	− 0.948	0.39	0.10	1.58	0.186
Weight	0.209	1.23	0.72	2.12	0.452
Height	− 0.073	0.93	0.55	1.58	0.788
Down syndrome	− 0.041	0.96	0.28	3.35	0.949
Main cardiac anomaly, AVSD vs. VSD	− 0.236	0.79	0.23	2.73	0.709
Peripheral oxygen saturation	− 0.207	0.81	0.49	1.36	0.433
Pulmonary/systemic blood flow ratio	− 0.077	0.93	0.54	1.59	0.782
Pulmonary venous flow (VTI)	− 0.017	0.98	0.58	1.67	0.950
TAPSE	− 0.531	0.59	0.32	1.07	0.084
CPB time	− 0.075	0.93	0.54	1.59	0.785
Pre-CPB mean pulmonary artery pressure	0.424	1.53	0.86	2.72	0.149
Pre-CPB pulmonary/systemic mean arterial pressure ratio	0.059	1.06	0.62	1.83	0.832
Post-CPB mean pulmonary artery pressure	0.789	2.20	1.18	4.11	0.013
Post-CPB mean systemic artery pressure	− 0.773	0.46	0.24	0.89	0.021
Post-CPB pulmonary/systemic mean arterial pressure ratio	0.965	2.62	1.32	5.20	0.006
Early postoperative PAP/SAP > 0.40	2.025	7.58	1.84	31.28	0.005
Presence of respiratory viral genomes in the trachea	1.055	2.87	0.83	9.99	0.097

All numeric variables were analyzed as quartiles

*AVSD* atrioventricular septal defect, *CPB* cardiopulmonary bypass, *PAP/SAP* pulmonary/systemic mean arterial pressure ratio, *TAPSE* tricuspid annular plane systolic excursion, *VSD* ventricular septal defect, *VTI* velocity–time integral

### Hemodynamics after discharge from the hospital

**Fig. 4:**
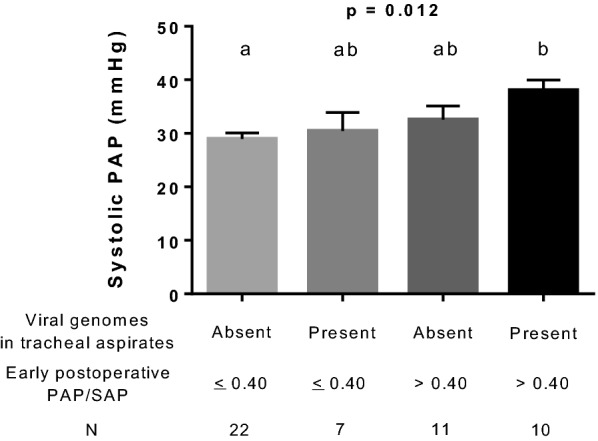
Interactive influence of respiratory viral genomes and early postoperative hemodynamics (pulmonary/systemic mean arterial pressure ratio, PAP/SAP) on post-hospitalization pulmonary arterial pressure. Shown are the results of patients for whom it was possible to analyze viral genetic material in tracheal aspirates and systolic pulmonary arterial pressure on 6-month postoperative echocardiogram. Data were analyzed using the general linear model and results are presented as means with SE. Groups not sharing the same letter were different at post hoc multiple comparisons

Fifty-eight individuals were discharged from the hospital and were seen in outpatient medical settings 6 months after operation. In 55 subjects, there was an adequate tricuspid regurgitation jet Doppler signal on 6-month postoperative echocardiogram allowing for the estimation of systolic pulmonary arterial pressure. While analyzing factors and covariates that were recorded perioperatively, we observed that the presence of viral genomes in tracheal aspirates and early postoperative PAP/SAP interactively influenced post-hospitalization levels of pulmonary artery pressure (Fig. 4).

## Discussion

Viruses are the leading cause of respiratory disorders and lung cell injury. This is particularly so in the pediatric population [25–27]. In the setting of cardiac surgery, respiratory abnormalities initiated preoperatively in the course of viral diseases may be further exacerbated by the systemic inflammatory reaction elicted by CPB [28] placing patients at greater risk for poor postoperative outcomes [8–10, 29, 30]. To the best of our knowledge, the present study represents the first initiative to investigate the relationship between respiratory viruses and the behavior of the pulmonary circulation after surgery for congenital cardiac shunts. Our findings suggest that respiratory viruses might have somehow contributed to the development of pulmonary vascular changes preoperatively, thus increasing the predisposition to postoperative pulmonary hypertension. Post-CPB inflammatory reaction seemed to play a role as a triggering factor. Altered postoperative hemodynamics accounted for clinical events in the intensive care unit with longer times of mechanical ventilation. Finally, respiratory viruses and early postoperative hemodynamics played an interactive role in determining the level of pulmonary artery pressure after discharge from the hospital. Obtainment of tracheal aspirates was crucial for demonstrating the aforementioned associations. The presence of genetic material for respiratory viruses in nasopharynx was by no means predictive of any hemodynamic or clinical events.

Some of our patients had respiratory viral infections in their clinical history, others did not. The question might be raised if respiratory viruses remain active in asymptomatic individuals. In addition, because different pathogens were detected in the study population, it would be worth discovering which ones are capable of infecting the distal lung as to cause relevant changes in small airways and eventually vessels. Although these are difficult points to address in the context of the present study, preliminary thoughts can be formulated with regard to rhinovirus, the most prevalent agent in our cohort. Rhinoviruses are known for their chronic behavior in many instances and their ability to alter the biology of peripheral lung cells. Although infections are usually mild and limited to the upper respiratory tract, there is growing evidence that they can induce lower respiratory tract pathology with severe pulmonary and extrapulmonary complications in adults and children [31, 32]. In the pediatric population, rhinovirus infections have been linked clinically with serious lower airway illnesses including asthma exacerbation, cystic fibrosis, bronchitis, bronchiolitis, pneumonia and croup [33–36]. Although most rhinovirus serotypes replicate optimally at 33 °C, some strains can actually replicate well at 37 °C and infect lower airways in humans [37]. Of special interest in terms of pulmonary vascular biology, rhinovirus-infected lung vascular endothelial cells generate several inflammatory and cytopathic responses [38]. Rhinovirus-infected bronchial epithelial cells express inflammatory mediators including IP-10 which was shown to be involved in vascular smooth muscle cell migration and proliferation [11, 15, 39]. Furthermore, type 2 immune response which is induced during rhinovirus infections [40, 41] was shown to play a central role in pulmonary vascular remodeling [13, 42]. In the present study, we were able to demonstrate the hypertensive behavior of the pulmonary circulation for the specific subgroup of rhinovirus carriers. Although the study was not sufficiently powered to demonstrate the individual role of other viruses, we speculate that some might be involved. For example, human bocavirus, the second most prevalent agent in our cohort was shown to persist in the infected host, induce the expression of cytokines and growth factors and elict Th1 and Th2 immune responses [43, 44].

In our patients, the inflammatory response to surgery under CPB was characterized by an increase in the number of circulating monocytes, a marked increase in neutrophil to lymphocyte ratio, a decrease in platelet count and changes in serum levels of several cytokines and related proteins. The inflammatory reaction seemed to explain the hypertensive postoperative behavior of the pulmonary circulation at least in part. While it is known that multiple mechanisms are involved in pulmonary vasoconstriction and hypertension in acute conditions [45], it is worth commenting on the role of some inflammatory mediators. IL-1, IL-6 and TNF-α protein levels were shown to be closely associated with PI3K/Akt signaling pathway activation in early stages of monocrotaline-induced pulmonary hypertension in rats [46]. The chemokine-like cytokine MIF, a central element in innate and adaptative immunity was shown to enhance pulmonary vasoconstriction induced by hypoxia and potentiate constriction pre-evoked by agonists in isolated pulmonary artery rings [47]. Although the chemokine MCP-1 has been shown to play an important pathophysiological role in pulmonary vascular remodeling [48–50], its involvement in acute pulmonary vasoconstriction is probably indirect. MCP-1 is expressed during endothelial cell activation, attracts inflammatory cells and enhances the release of several other mediators of inflammation [51]. Interestingly, MCP-1 was shown to play a role in the recovery of monocyte function after pediatric cardiac surgery along with IL-1RA and IL-10 [52]. In the present study, the increase in IL-1 activity following CPB was inferred from the ~ tenfold increase in serum level of IL-1RA. Because of the spare receptor phenomenon, large quantities of IL-1RA are required to functionally inhibit the biological effects of negligible amounts of IL-1 [53]. Our results showed that preoperative exposure to respiratory viruses and postoperative overexpression of IL-1/IL-1RA, MIF and MCP-1 acted as combined risk factors for postoperative pulmonary hypertension. Other cytokines (e.g., IL-6 and IL-21) probably played a role as judged by the data presented in Table 5. Furthermore, because the level of several inflammatory proteins changed significantly following surgery, as shown in Table 4, the spectrum of mediators with relevant effects on the pulmonary and systemic circulation and the heart may be even wider.

We also investigated factors that have been classically linked to postoperative pulmonary hypertension in this population. While patient’s age (maximum of 35 months in the study) did not seem to influence postoperative pulmonary hemodynamics, the presence of Down syndrome may have played an indirect role via the complexity of cardiac anomaly (atrioventricular septal defect in 61.5% of cases) with longer surgical times (CPB duration) and presumably, more pronounced inflammatory response. Peripheral oxygen saturation was found to be an important predictor of postoperative hemodynamics. This was probably due to the fact that oxygen saturation reflected the status of the pulmonary circulation at baseline. In fact, bedside oxygen saturation was negatively correlated with pre-CPB pulmonary artery pressure measured directly in the operating room. Although oxygen saturation did not correlate with the presence/absence of viral genomes in the airways, we cannot totally exclude chronic airway abnormalities as a cause for lower-than-normal oxygen saturation levels in some individuals. Upper airway obstructions may have contributed to imbalances between ventilation and perfusion especially in patients with Down syndrome. Bidirectional shunting within the cardiac chambers as a result of heightened pulmonary vascular resistance may also have contributed to systemic oxygen desaturation in some cases.

## Conclusion

We showed an association between the presence of respiratory viral genomes in the trachea and the hypertensive behavior of the pulmonary circulation in pediatric patients undergoing surgery for congenital cardiac septal defects, thus suggesting a pathophysiological role for respiratory viruses on the development of pulmonary vascular abnormalities. Because we did not investigate if patients were still carriers for viruses after operation (study limitation), we can only speculate that new biological events initiated at the time of surgery involving inflammation and persistence of viral activity might partly explain the hemodynamic alterations that were still present in some individuals after discharge from the hospital. Further research is needed to clarify such relationships.

## Data Availability

The data used to support the conclusion of the present study correspond to the project FAPESP “Respiratory viruses, systemic inflammation and postoperative pulmonary hypertension in congenital heart disease”, and are available from the corresponding author upon request.

## Abbreviations

CPB: Cardiopulmonary bypass
ECG: Electrocardiogram
TAPSE: Tricuspid annular plane systolic excursion
PAP/SAP: Pulmonary/systemic mean arterial pressure ratio
RT-PCR: Real-time polymerase chain reaction
upi: Units of pixel intensity
C5/C5a: Complement component 5/5a
CD40L: CD40 ligand
G-CSF: Granulocyte colony-stimulating factor
GM–CSF: Granulocyte–macrophage colony-stimulating factor
GROα: Growth-regulated oncogene alpha
I-309: Human CC chemokine I-309
ICAM-1: Intercellular adhesion molecule-1
IFN-γ: Interferon gamma
IL: Interleukins 1 alpha, 1 beta, 2, 4, 5, 6, 8, 10, 12, 13, 16, 17, 17E, 18, 21, 27 and 32 alpha
IL-1RA: Interleukin-1 receptor antagonist
IP-10: Interferon gamma-induced protein-10
I-TAC: Interferon-inducible T cell alpha chemoattractant
MCP-1: Monocyte chemoattractant protein-1
MIF: Macrophage migration inhibitory factor
MIP-1α/β: Macrophage inflammatory protein-1 alpha/beta
RANTES: Regulated on activation, normal T cell expressed and secreted
Serpin E1: Plasminogen activator inhibitor-1
SDF-1: Stromal cell-derived factor-1
TNF-α: Tumor necrosis factor alpha
sTREM-1: Soluble triggering receptor expressed on myeloid cells-1

## References

[1] Gorenflo M, Gu H, Xu Z (2010). Peri-operative pulmonary hypertension in paediatric patients: current strategies in children with congenital heart disease. Cardiology.

[2] Kaestner M, Schranz D, Warnecke G, Apitz C, Hansmann G, Miera O (2016). Pulmonary hypertension in the intensive care unit. Expert consensus statement on the diagnosis and treatment of paediatric pulmonary hypertension. The European Paediatric Pulmonary Vascular Disease Network, endorsed by ISHLT and DGPK. Heart.

[3] Marwali EM, Rayhan M, Roebiono PS (2021). Nitroglycerin inhalation for acute treatment of pulmonary arterial hypertension in children with congenital heart disease. Cardiol Young.

[4] D'Alto M, Mahadevan VS (2012). Pulmonary arterial hypertension associated with congenital heart disease. Eur Respir Rev.

[5] Adatia I, Beghetti M (2011). Pulmonary hypertension and postoperative congenital heart disease. Pediatric pulmonary hypertension.

[6] Falcone N (2001). Hipertensión pulmonar en cirugía cardíaca pediátrica [Pulmonary hypertension in pediatric heart surgery]. Rev Esp Anestesiol Reanim.

[7] Sugawara Y, Mizuno Y, Oku S, Goto T (2019). Effects of vasopressin during a pulmonary hypertensive crisis induced by acute hypoxia in a rat model of pulmonary hypertension. Br J Anaesth.

[8] Spaeder MC, Carson KA, Vricella LA, Alejo DE, Holmes KW (2011). Impact of the viral respiratory season on postoperative outcomes in children undergoing cardiac surgery. Pediatr Cardiol.

[9] Moynihan K, Barlow A, Alphonso N, Anderson B, Johnson J, Nourse C (2017). Impact of viral respiratory pathogens on outcomes after pediatric cardiac surgery. Pediatr Crit Care Med.

[10] Li X, Wang X, Li S, Zeng M, Li D (2020). Viral respiratory infection, a risk in pediatric cardiac surgery: a propensity-matched analysis. Pediatr Crit Care Med.

[11] Wang X, Yue TL, Ohlstein EH, Sung CP, Feuerstein GZ (1996). Interferon-inducible protein-10 involves vascular smooth muscle cell migration, proliferation, and inflammatory response. J Biol Chem.

[12] Chung KF, Barnes PJ (1999). Cytokines in asthma. Thorax.

[13] Daley E, Emson C, Guignabert C, de Waal MR, Louten J, Kurup VP (2008). Pulmonary arterial remodeling induced by a Th2 immune response. J Exp Med.

[14] Zeng R, Li C, Li N, Wei L, Cui Y (2011). The role of cytokines and chemokines in severe respiratory syncytial virus infection and subsequent asthma. Cytokine.

[15] Cakebread JA, Haitchi HM, Xu Y, Holgate ST, Roberts G, Davies DE (2014). Rhinovirus-16 induced release of IP-10 and IL-8 is augmented by Th2 cytokines in a pediatric bronchial epithelial cell model. PLoS ONE.

[16] Shelfoon C, Shariff S, Traves SL, Kooi C, Leigh R, Proud D (2016). Chemokine release from human rhinovirus-infected airway epithelial cells promotes fibroblast migration. J Allergy Clin Immunol.

[17] Shariff S, Shelfoon C, Holden NS, Traves SL, Wiehler S, Kooi C (2017). Human rhinovirus infection of epithelial cells modulates airway smooth muscle migration. Am J Respir Cell Mol Biol.

[18] Hislop AA (2002). Airway and blood vessel interaction during lung development. J Anat.

[19] Hansmann G, Koestenberger M, Alastalo TP, Apitz C, Austin ED, Bonnet D (2019). 2019 updated consensus statement on the diagnosis and treatment of pediatric pulmonary hypertension: the European Pediatric Pulmonary Vascular Disease Network (EPPVDN), endorsed by AEPC, ESPR and ISHLT. J Heart Lung Transplant.

[20] Souza MFS, Penha JG, Maeda NY, Galas FRBG, Abud KCO, Carvalho ES (2022). Postoperative pulmonary hemodynamics and systemic inflammatory response in pediatric patients undergoing surgery for congenital heart defects. Mediat Inflamm.

[21] Gaies MG, Jeffries HE, Niebler RA, Pasquali SK, Donohue JE (2014). Vasoactive-inotropic score is associated with outcome after infant cardiac surgery: an analysis from the pediatric cardiac critical care consortium and virtual PICU system registries. Pediatr Crit Care Med.

[22] Ribeiro ZV, Tsutsui JM, Miranda RDA, Mohry S, Mathias W, Lopes AA (2010). Ecocardiografia-Doppler e parâmetros hemodinâmicos em cardiopatias congênitas com hiperfluxo pulmonar [Doppler echocardiography and hemodynamic parameters in congenital heart disease with increased pulmonary flow]. Arq Bras Cardiol.

[23] Rivera IR, Mendonça MA, Andrade JL, Moises V, Campos O, Silva CC (2013). Pulmonary venous flow index as a predictor of pulmonary vascular resistance variability in congenital heart disease with increased pulmonary flow: a comparative study before and after oxygen inhalation. Echocardiography.

[24] Koestenberger M, Ravekes W, Everett AD, Stueger HP, Heinzl B, Gamillscheg A (2009). Right ventricular function in infants, children and adolescents: reference values of the tricuspid annular plane systolic excursion (TAPSE) in 640 healthy patients and calculation of z-score values. J Am Soc Echocardiogr.

[25] Tahamtan A, Besteman S, Samadizadeh S, Rastegar M, Bont L, Salimi V (2021). Neutrophils in respiratory syncytial virus infection: from harmful effects to therapeutic opportunities. Br J Pharmacol.

[26] Connors TJ, Ravindranath TM, Bickham KL, Gordon CL, Zhang F, Levin B (2016). Airway CD8(+) T cells are associated with lung injury during infant viral respiratory tract infection. Am J Respir Cell Mol Biol.

[27] Hosakote YM, Liu T, Castro SM, Garofalo RP, Casola A (2009). Respiratory syncytial virus induces oxidative stress by modulating antioxidant enzymes. Am J Respir Cell Mol Biol.

[28] Inoue N, Oka N, Kitamura T, Shibata K, Itatani K, Tomoyasu T (2013). Neutrophil elastase inhibitor sivelestat attenuates perioperative inflammatory response in pediatric heart surgery with cardiopulmonary bypass. Int Heart J.

[29] Delgado-Corcoran C, Witte MK, Ampofo K, Castillo R, Bodily S, Bratton SL (2014). The impact of human rhinovirus infection in pediatric patients undergoing heart surgery. Pediatr Cardiol.

[30] Giffin NA, Guerra G, Robinson J, Joynt C, Rebeyka I, Ben SV (2021). Impact of early surgical correction or palliation of congenital heart defects in infants with symptomatic viral respiratory tract infections in the current era. JTCVS Open.

[31] Papadopoulos NG, Bates PJ, Bardin PG, Papi A, Leir SH, Fraenkel DJ (2000). Rhinoviruses infect the lower airways. J Infect Dis.

[32] To KKW, Yip CCY, Yuen KY (2017). Rhinovirus—from bench to bedside. J Formos Med Assoc.

[33] Kieninger E, Singer F, Tapparel C, Alves MP, Latzin P, Tan HL (2013). High rhinovirus burden in lower airways of children with cystic fibrosis. Chest.

[34] Hayden FG (2004). Rhinovirus and the lower respiratory tract. Rev Med Virol.

[35] Jackson DJ, Gern JE (2022). Rhinovirus infections and their roles in asthma: etiology and exacerbations. J Allergy Clin Immunol Pract.

[36] Cox DW, Le Souëf PN (2014). Rhinovirus and the developing lung. Paediatr Respir Rev.

[37] Papadopoulos NG, Sanderson G, Hunter J, Johnston SL (1999). Rhinoviruses replicate effectively at lower airway temperatures. J Med Virol.

[38] Likońska A, Gawrysiak M, Gajewski A, Klimczak K, Michlewska S, Szewczyk R (2022). Human lung vascular endothelium may limit viral replication and recover in time upon the infection with rhinovirus HRV16. APMIS.

[39] Wark PA, Bucchieri F, Johnston SL, Gibson PG, Hamilton L, Mimica J (2007). IFN-gamma-induced protein 10 is a novel biomarker of rhinovirus-induced asthma exacerbations. J Allergy Clin Immunol.

[40] Hasegawa K, Hoptay CE, Harmon B, Celedón JC, Mansbach JM, Piedra PA (2019). Association of type 2 cytokines in severe rhinovirus bronchiolitis during infancy with risk of developing asthma: a multicenter prospective study. Allergy.

[41] Southworth T, Pattwell C, Khan N, Mowbray SF, Strieter RM, Erpenbeck VJ (2020). Increased type 2 inflammation post rhinovirus infection in patients with moderate asthma. Cytokine.

[42] Chen G, Zuo S, Tang J, Zuo C, Jia D, Liu Q (2018). Inhibition of CRTH2-mediated Th2 activation attenuates pulmonary hypertension in mice. J Exp Med.

[43] Chung JY, Han TH, Kim JS, Kim SW, Park CG, Hwang ES (2008). Th1 and Th2 cytokine levels in nasopharyngeal aspirates from children with human bocavirus bronchiolitis. J Clin Virol.

[44] Khalfaoui S, Eichhorn V, Karagiannidis C, Bayh I, Brockmann M, Pieper M (2016). Lung infection by human bocavirus induces the release of profibrotic mediator cytokines in vivo and in vitro. PLoS ONE.

[45] Revercomb L, Hanmandlu A, Wareing N, Akkanti B, Karmouty-Quintana H (2021). Mechanisms of pulmonary hypertension in acute respiratory distress syndrome (ARDS). Front Mol Biosci.

[46] Tang C, Luo Y, Li S, Huang B, Xu S, Li L (2021). Characteristics of inflammation process in monocrotaline-induced pulmonary arterial hypertension in rats. Biomed Pharmacother.

[47] Zhang B, Luo Y, Liu ML, Wang J, Xu DQ, Dong MQ (2012). Macrophage migration inhibitory factor contributes to hypoxic pulmonary vasoconstriction in rats. Microvasc Res.

[48] Sanchez O, Marcos E, Perros F, Fadel E, Tu L, Humbert M (2007). Role of endothelium-derived CC chemokine ligand 2 in idiopathic pulmonary arterial hypertension. Am J Respir Crit Care Med.

[49] Ikeda Y, Yonemitsu Y, Kataoka C, Kitamoto S, Yamaoka T, Nishida K (2002). Anti-monocyte chemoattractant protein-1 gene therapy attenuates pulmonary hypertension in rats. Am J Physiol Heart Circ Physiol.

[50] Gosemann JH, Friedmacher F, Hofmann A, Zimmer J, Kuebler JF, Rittinghausen S (2018). Prenatal treatment with rosiglitazone attenuates vascular remodeling and pulmonary monocyte influx in experimental congenital diaphragmatic hernia. PLoS ONE.

[51] Singh S, Anshita D, Ravichandiran V (2021). MCP-1: function, regulation, and involvement in disease. Int Immunopharmacol.

[52] Justus G, Walker C, Rosenthal LM, Berger F, Miera O, Schmitt KRL (2019). Immunodepression after CPB: cytokine dynamics and clinics after pediatric cardiac surgery—a prospective trial. Cytokine.

[53] Arend WP (2002). The balance between IL-1 and IL-1Ra in disease. Cytokine Growth Factor Rev.

